# Effect of inhibition of CBP-coactivated β-catenin-mediated Wnt signalling in uremic rats with vascular calcifications

**DOI:** 10.1371/journal.pone.0201936

**Published:** 2018-08-03

**Authors:** Eva Gravesen, Anders Nordholm, Maria Mace, Marya Morevati, Estrid Høgdall, Carsten Nielsen, Andreas Kjær, Klaus Olgaard, Ewa Lewin

**Affiliations:** 1 Department of Clinical Medicine, Faculty of Health Sciences, University of Copenhagen, Denmark; 2 Nephrological Department P, Rigshospitalet, Copenhagen, Denmark; 3 Nephrological Department B, Herlev Hospital, Copenhagen, Denmark; 4 Molecular Unit, Department of Pathology, Herlev Hospital, Copenhagen, Denmark; 5 Cluster for molecular imaging, Department of Biomedical Sciences and Department of Clinical Physiology, Nuclear Medicine & PET, Rigshospitalet and University of Copenhagen, Denmark; University Medical Center Utrecht, NETHERLANDS

## Abstract

Uremic vascular calcification is a regulated cell-mediated process wherein cells in the arterial wall transdifferentiate to actively calcifying cells resulting in a process resembling bone formation. Wnt signalling is established as a major driver for vessel formation and maturation and for embryonic bone formation, and disturbed Wnt signalling might play a role in vascular calcification. ICG-001 is a small molecule Wnt inhibitor that specifically targets the coactivator CREB binding protein (CBP)/β-catenin-mediated signalling. In the present investigation we examined the effect of ICG-001 on vascular calcification in uremic rats. Uremic vascular calcification was induced in adult male rats by 5/6-nephrectomy, high phosphate diet and alfacalcidol. The presence of uremic vascular calcification in the aorta was associated with induction of gene expression of the Wnt target gene and marker of proliferation, cyclinD1; the mediator of canonical Wnt signalling, β-catenin and the matricellular proteins, fibronectin and periostin. Furthermore, genes from fibrosis-related pathways, TGF-β and activin A, as well as factors related to epithelial-mesenchymal transition, snail1 and vimentin were induced. ICG-001 treatment had significant effects on gene expression in kidney and aorta from healthy rats. These effects were however limited in uremic rats, and treatment with ICG-001 did not reduce the Ca-content of the uremic vasculature.

## Introduction

Patients with chronic kidney disease (CKD) suffer from severe vascular calcification (VC) [1] and excess cardiovascular morbidity and mortality [2, 3] that are not fully explained by the presence of traditional risk factors such as hypertension, diabetes and old age [4]. Disorders in the mineral homeostasis and bone are today recognized as having a fundamental role in the cardiovascular complications of CKD. This syndrome has been named CKD-mineral and bone disorder (CKD-MBD) [5]. Although VC is associated with mineral disturbances and the proinflammatory uremic state, the precise mechanisms behind the development of uremic VC are still unknown [6]. Research suggests that VC is a product of highly regulated cell-mediated processes wherein cells in the arterial wall transdifferentiate and become actively calcifying cells, resulting in a process resembling bone formation [7]. Wnt signalling is established as a major driver for vessel formation and maturation [8] and for embryonic bone formation [9]. Although Wnt signalling by and large is silenced in the mature vascular system, it is involved in adult bone homeostasis, increasing osteoblast maturation which results in increased bone formation [10]. Research suggests, that reactivation of Wnt signalling might be involved in different organ diseases and cancer forms [11] as well as in development of fibrosis and epithelial-mesenchymal transition (EMT) [12, 13]. Further, evidence from several research groups indicates that both canonical (β-catenin-dependent) and non-canonical Wnt signalling are involved in such cardiovascular diseases as atherosclerosis and VC [14]. As VC resembles bone formation it is an interesting thought that disturbed Wnt signalling might play a role in the pathogenesis of VC. In a previous investigation we used high throughput RNA deep sequencing (RNAseq) on the calcified aorta from uremic rats to study the transcriptional changes that occur in the aorta between the normal and the uremic, calcified condition [15]. In the RNAseq study, we found that the expression of a number of Wnt modulators, Wnt-inducible genes and Wnt ligands was altered (S1 Table). Induction of canonical Wnt signalling can result in both proliferation and differentiation. This divergence might be explained by the binding of β-catenin to one of two different transcriptional coactivators, CBP and p300 [16]. In the RNAseq investigation we found increased expression of Ccnd1 (coding for cyclinD1) and decreased expression of Jun in the calcified aorta from uremic rats. CyclinD1 is a marker of increased proliferation that might be a target of CBP-coactivated β-catenin signalling [17] whereas Jun might be a target of p300-coactivated signalling [18]. Our previous results therefore may suggest a potential involvement of CBP-coactivated Wnt signalling in the uremic VC.

The Wnt inhibitor ICG-001 has previously been shown to inhibit development of fibrosis in several organs, such as lungs and kidneys [19, 20]. It specifically binds to the β-catenin coactivator CREB binding protein (CBP) at the β-catenin binding site, and thereby hinders CBP/β-catenin-mediated signalling [21, 22].

In the present investigation we examined the effects of ICG-001 on the vasculature, kidneys and bone in uremic rats with focus on the potential reversibility of uremic vascular calcification by inhibition of the CBP-coactivated β-catenin-mediated Wnt signalling.

## Results

### Induction of CRF and associated disturbances in mineral metabolism

**Fig 1:**
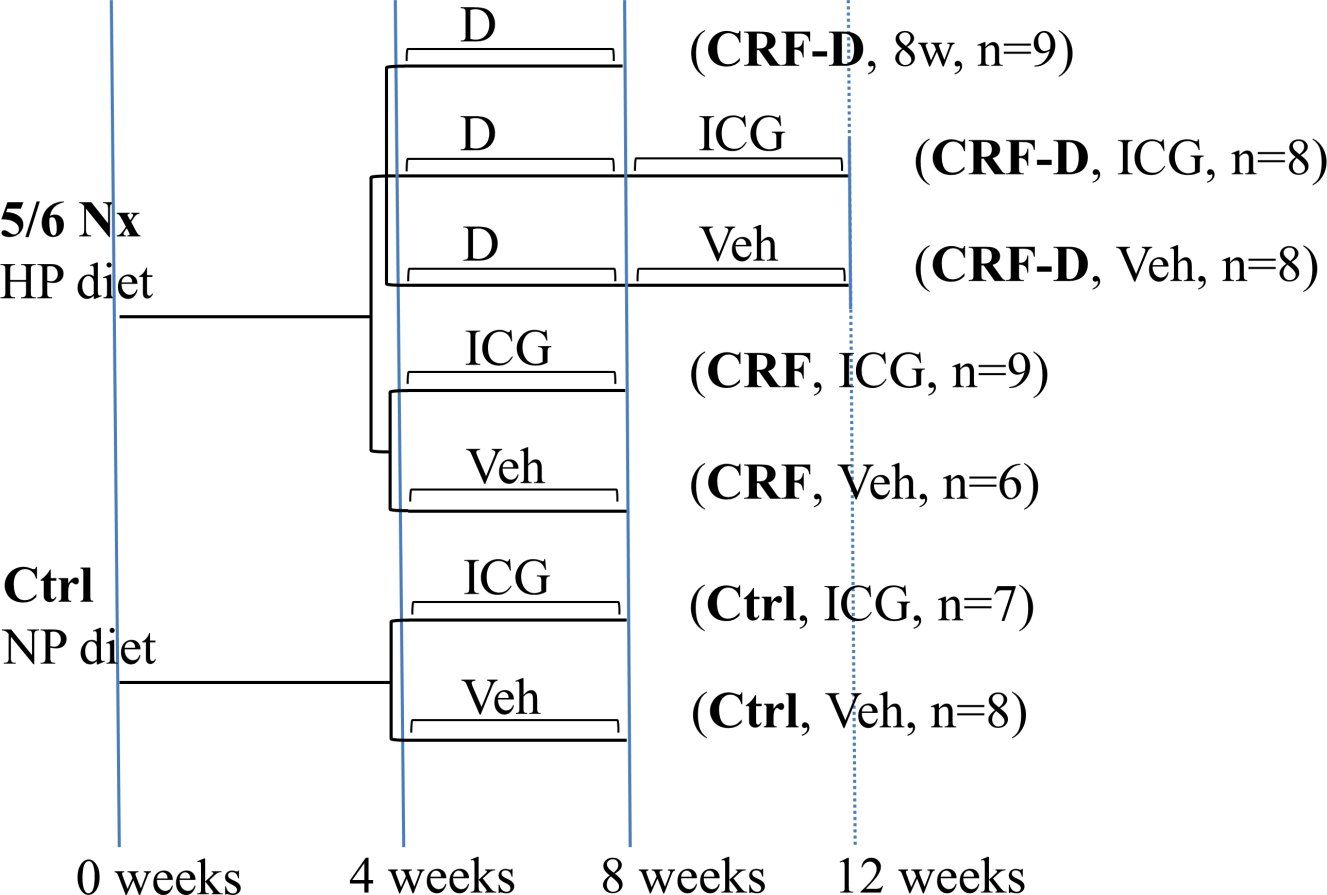
Experimental design. 5/6 Nx = 5/6 nephrectomy, Ctrl = control, CRF = chronic renal failure, HP = high phosphate, NP = normal phosphate, D = alfacalcidol, ICG = ICG-001, Veh = Vehicle, 8w = 8weeks. After four weeks of uremia CRF rats were allocated to five experimental groups according to weight and plasma urea. CRF-D rats were treated with alfacalcidol to establish VC. At eight weeks the alfacalcidol was stopped and subsequently rats received daily injections of either ICG-001 or vehicle for four weeks. Rats were sacrificed after 12 weeks of uremia. A group of alfacalcidol treated CRF rats was sacrificed at 8 weeks to examine the alfacalcidol-induced mineral metabolism disturbances at baseline. CRF rats not treated with alfalcalcidol and Ctrl rats were allocated to four weeks of either ICG-001 or vehicle and sacrificed after 8 weeks.

Chronic renal failure (CRF) was induced in adult male rats by 5/6-nephrectomy and ICG-001 was administered daily as intraperitoneal (ip) injections in a treatment protocol after the induction of vascular calcification with high phosphate diet and alfacalcidol (CRF-D) as outlined in Fig 1. A control group of uremic rats that were not treated with alfalcalcidol (CRF) was used to examine the potential effects of ICG-001 on uremia and on the fibrotic remnant kidney of the 5/6-nephrectomy model, and a control group of normal age-matched rats (Ctrl) were kept in parallel and allocated to either ICG-001 or vehicle.

**Fig 2:**
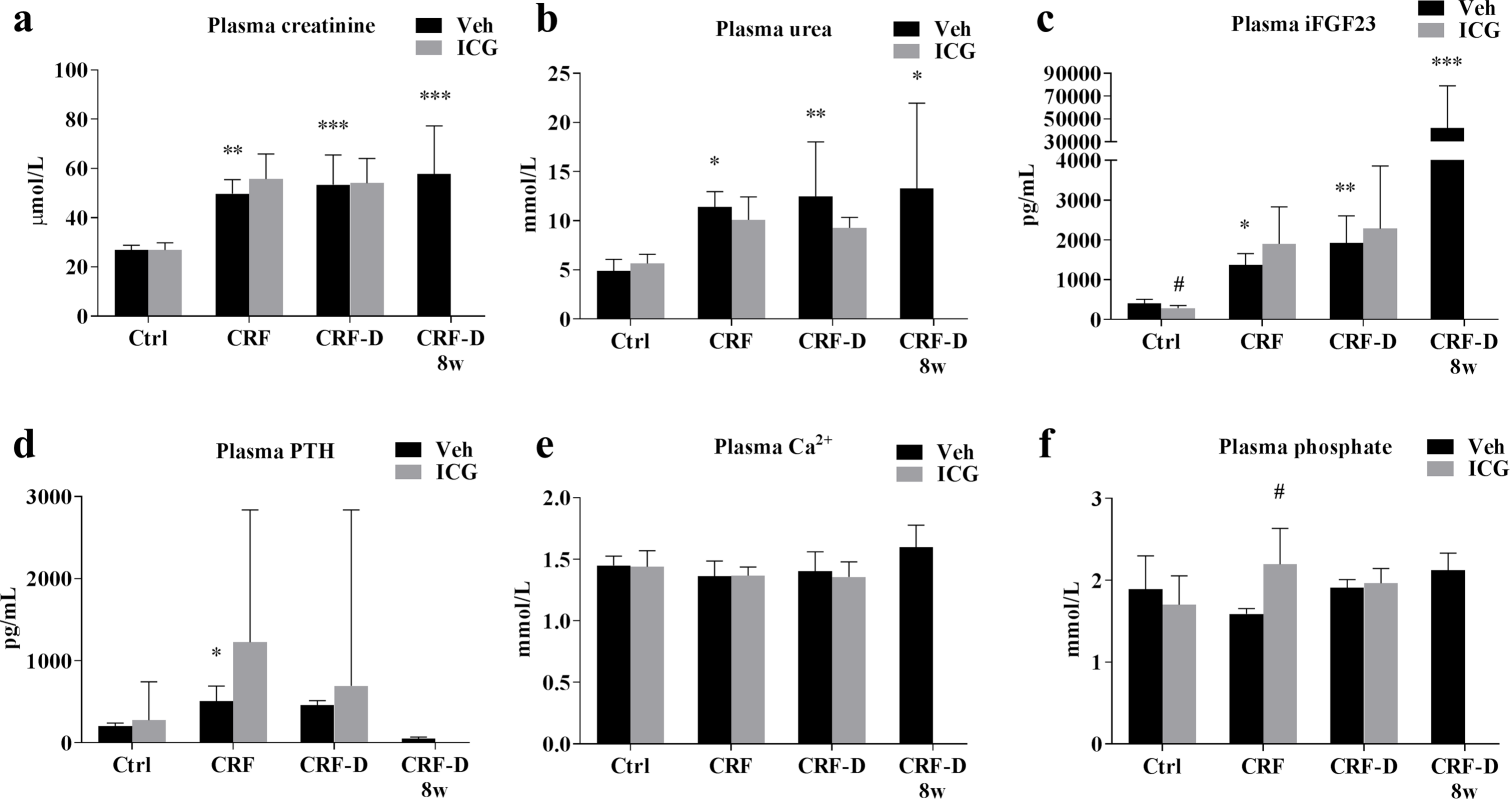
Plasma biochemistry. Plasma levels of (**a**) creatinine, (**b**) urea, (**c**) intact fibroblast growth factor 23 (iFGF23), (**d**) PTH, (**e**) Ca^2+^ and (**f**) phosphate measured at sacrifice. CRF and CRF-D rats had a mild to moderate reduction in kidney function with increased levels of plasma creatinine, urea, iFGF23 and PTH. In the CRF-D rats sacrificed at 8 weeks the iFGF23 levels were extremely elevated, PTH was suppressed, and a trend towards an increase in Ca^2+^ was seen. iFGF23 levels were slightly lower in ICG-001-administered Ctrl compared to vehicle-administered Ctrl and plasma phosphate was markedly higher in the ICG-001-administered CRF compared to the vehicle-administered CRF. Data is presented as mean ± SD, and PTH as median and interquartile range. n = 6–9. Vehicle-administered Ctrl, CRF and CRF-D rats were compared by one-way ANOVA and Dunnets multiple comparison with *P<0.05, **P<0.001 and ***P<0.0001 vs Ctrl. ICG-001- and vehicle-administered groups were compared using two-tailed t-test with ^#^P <0.05 vs vehicle.

Total plasma biochemistry and body weight is available in S2 Table. CRF and CRF-D rats had significantly increased plasma levels of urea, creatinine, parathyroid hormone (PTH) and intact fibroblast growth factor 23 (iFGF23) compared to Ctrl rats (Fig 2A–2D). In the group of CRF-D rats sacrificed at 8 weeks, the body weight was lower (P<0.05), total Ca was elevated (P<0.05), PTH was suppressed and FGF23 was extremely elevated (P<0.0001) compared to Ctrl rats (Fig 2C and 2D). A significant decrease in iFGF23 levels was seen in ICG-001-administered Ctrl rats compared to vehicle-administered Ctrl rats (P<0.05) (Fig 2C). A significantly higher mean plasma phosphate was seen in ICG-001-administered CRF rats compared to vehicle-administered CRF rats (P<0.05) (Fig 2F). No other significant differences were seen between ICG-001- and vehicle-administered rats.

### Effects of ICG-001 on kidney gene and protein expression

To ensure the bioavailability and effect of ICG-001, a short-term experiment was conducted wherein the effect of ICG-001 on kidney gene expression was examined in a model of acutely induced renal fibrosis, the unilateral ureteral obstruction (UUO) model. Three days of UUO was accompanied by a significant induction in the gene expression of fibronectin (Fn1), inhibin-βa (Inhba) and vimentin (Vim) in the obstructed kidney. Administration of ICG-001 abolished the expression of Fn1 and Inhba which was induced by the ureteral obstruction and reduced the expression of the Wnt target gene, Snai1 (S1 Fig).

As ICG-001 previously has been reported to have anti-fibrotic effects, the effects of ICG-001 on gene- and protein expression in the remnant kidney of the 5/6-Nx model was examined, as a reduction in kidney damage potentially might have indirect beneficial effects on the vasculature.

**Fig 3:**
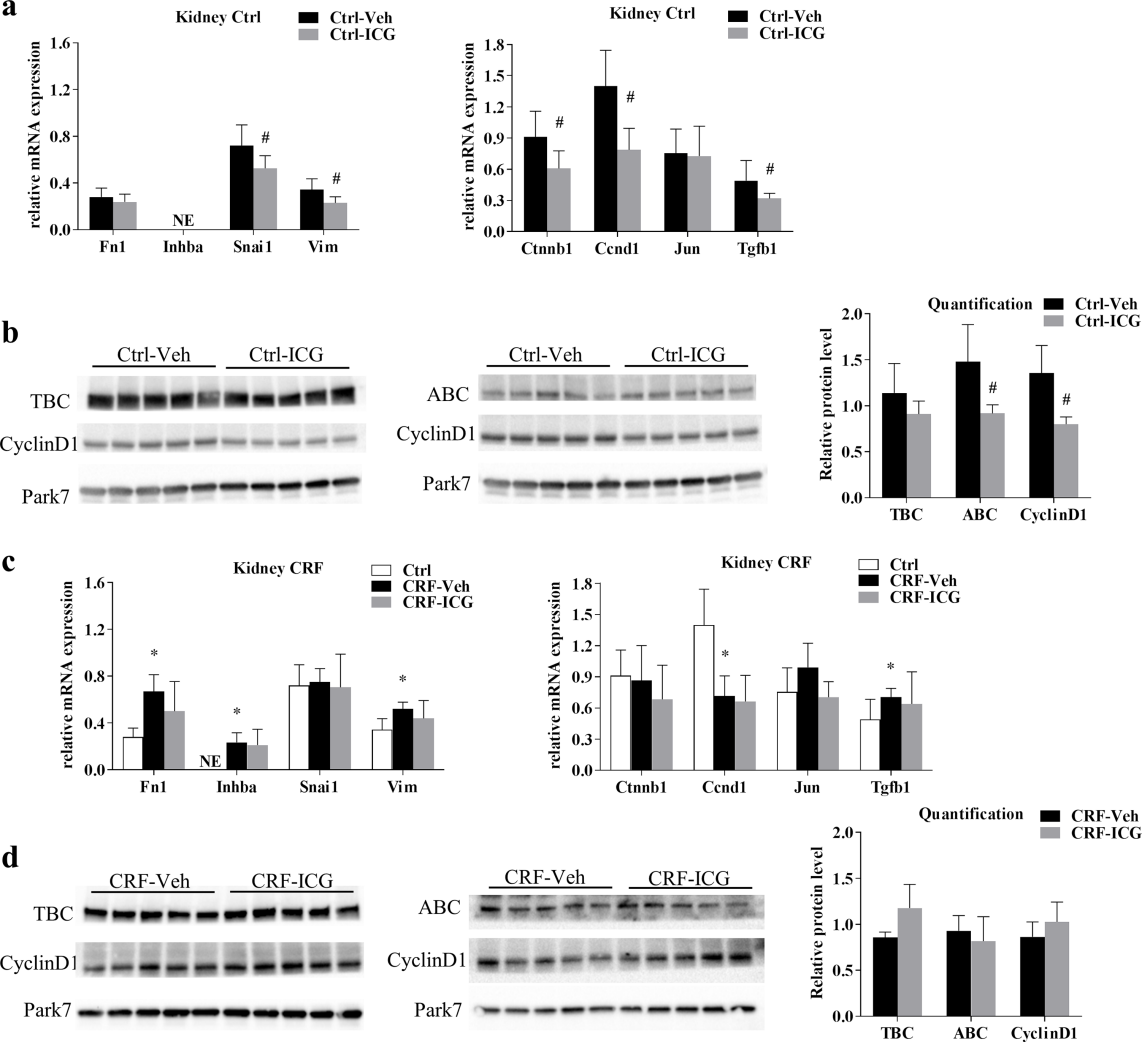
Kidney gene and protein expression. Kidney gene expression of factors related to fibrosis and EMT as well as Wnt signalling was examined in Ctrl rats (**a)** and in CRF rats (**c**). In Ctrl rats ICG-001 resulted in a decrease in the expression of Snai1 (snail1), Vim (vimentin), Ctnnb1 (β-catenin), Ccnd1 (cyclinD1) and Tgfb1 (TGF-β), while these effects were eliminated in CRF rats. Protein levels of total β-catenin (TBC), active β-catenin (ABC) and cyclinD1 were examined by WB in kidneys from Ctrl (**b**) and CRF rats (**d**). In accordance with the gene expression results, protein levels of active β-catenin and cyclinD1 were reduced in kidneys from ICG-001-administered Ctrl rats while no differences in protein levels were seen between ICG-001- and vehicle-administered CRF rats. Blots cropped from different parts of the same gel are presented together. Uncropped blots are provided in S2 Fig. Data is presented as mean ± SD. n = 6–9. *P<0.05 vs Ctrl by unpaired two-tailed t-test, ^#^P <0.05 vs vehicle by unpaired two-tailed t-test.

To examine the potential effects of ICG-001 on established kidney fibrosis, the gene expression of β-catenin (Ctnnb1), the target genes of Wnt/β-catenin; cyclinD1 (Ccnd1) and c-Jun (Jun) and factors related to EMT; snail1 (Snai1) and vimentin (Vim), as well as the extracellular matrix associated gene fibronectin (Fn1) and genes from the related pathways; TGF-β (Tgfb1) and activin A (Inhba) were all examined by qPCR in kidneys obtained from Ctrl and CRF rats. Fn1 and Vim were expressed at low levels and Inhba was not expressed in kidneys from Ctrl rats (Fig 3A). ICG-001 administration resulted in significant reductions in Snai1 and Vim expression (P<0.05), whereas Fn1 expression was unchanged by ICG-001 (Fig 3A). In Ctrl rats ICG-001 further resulted in a significant decrease in kidney gene expression of Ctnnb1, Ccnd1 and Tgfb1 (P<0.05) while the expression of Jun was unaffected (Fig 3A). The effect of ICG-001 on protein levels of cyclinD1, total β-catenin and active β-catenin was examined by Western blot. ICG-001-administered Ctrl rats had significantly lower levels of cyclinD1 and active β-catenin (P<0.05) (Fig 3B) compared to vehicle-administered Ctrl rats. In kidneys from CRF rats, the gene expression of Fn1, Inhba, Vim and Tgfb1 were higher (P<0.05) compared to Ctrl rats, while the gene expression of Ccnd1 was reduced (P<0.05). No differences in gene expression were seen between kidneys from ICG-001- and vehicle-administered CRF rats (Fig 3C). Likewise, no differences were seen in the protein levels of cyclinD1 or total and active β-catenin between kidneys from ICG-001- and vehicle-administered CRF rats examined by Western blot (Fig 3D).

### Effects of ICG-001 on aorta gene expression

**Fig 4:**
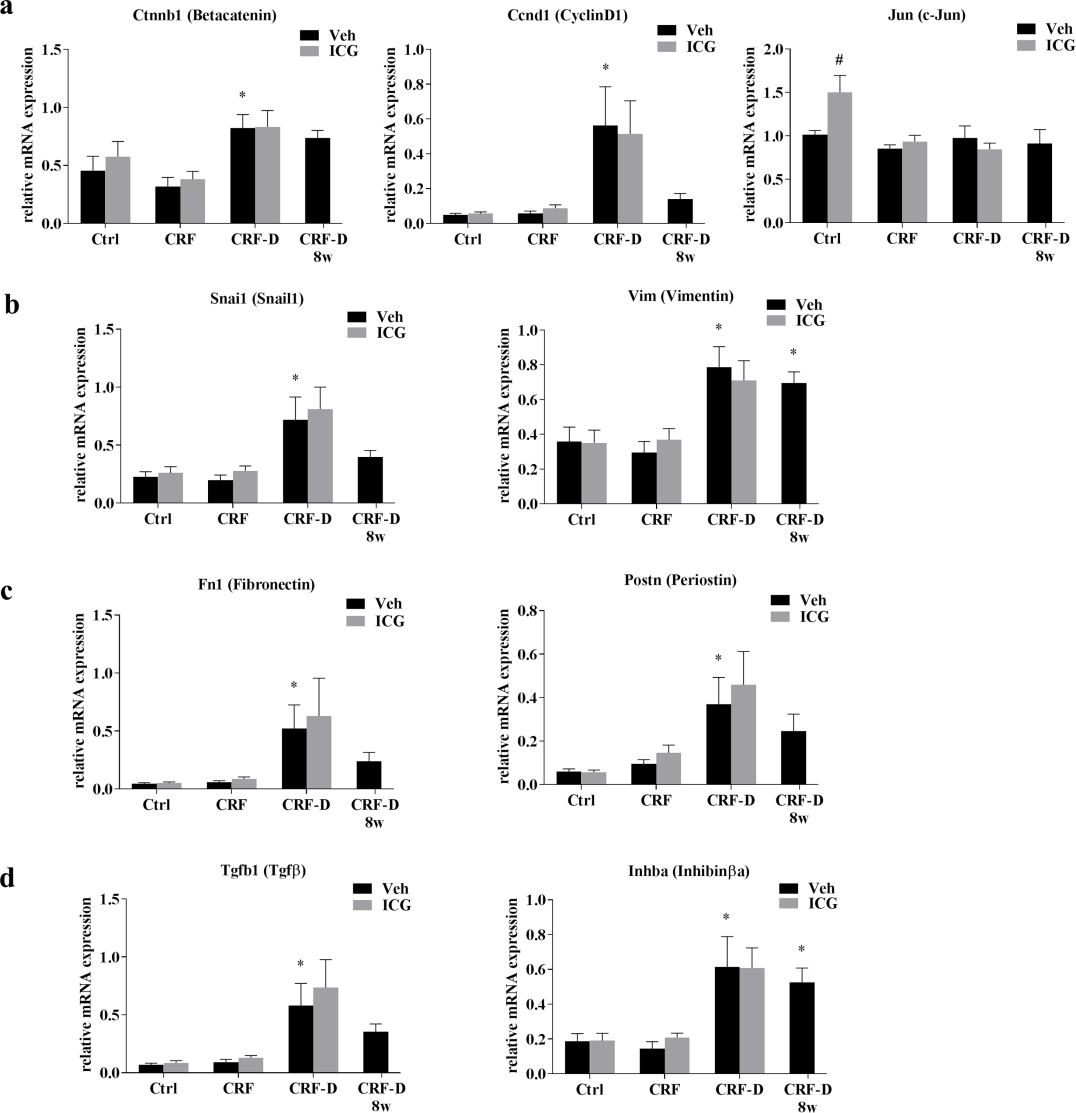
Aorta gene expression. Gene expression was examined in aortae from Ctrl, CRF and CRF-D rats by qPCR. The expression of genes related to (**a**) Wnt-signaling: Ctnnb1(β-catenin), Ccnd1 (cyclinD1), and Jun (c-Jun), (**b**) Epithelial-mesenchymal transition, EMT: Snai1 (snail1) and Vim (vimentin), **(c**) Extracellular matrix proteins: Fn1 (fibronectin) and Postn (periostin), and (**d**) genes from the TGF-β superfamily ligands: Tgfb1 (TGF-β) and Inhba (activin A) is shown. The expression of genes related to Wnt signalling; β-catenin and cyclinD1, and to EMT; snail1 and vimentin, and to extracellular matrix proteins; fibronectin and periostin as well as genes from the TGF-β superfamily ligands, TGF-β and activin A were all significantly induced in the aortae from CRF-D rats, while Jun was not changed. No differences in gene expression were seen between Ctrl and CRF rats, and the gene expression of cyclinD1, TGF-β and fibronectin was very low in these groups. The expression of Jun was higher in ICG-001-administered Ctrl compared to vehicle-administered Ctrl, while no other differences were noted between ICG-001- and vehicle-administered Ctrl, CRF or CRF-D rats. Data is presented as mean ± SD. n = 6–9. Vehicle-administered Ctrl, CRF and CRF-D rats were compared by one-way ANOVA and Dunnets multiple comparison with *P<0.05 vs Ctrl. ICG-001- and vehicle-treated groups were compared using two-tailed t-test with ^#^P <0.05 vs vehicle.

Aorta gene expression of genes related to Wnt signalling Ctnnb1, Ccnd1 and Jun (Fig 4A), factors related to epithelial-mesenchymal transition Snai1 and Vim (Fig 4B) as well as extracellular matrix proteins Fn1 and Postn (Fig 4C) and ligands from the TGF-β superfamily Tgfb1 and Inhba (Fig 4D) were examined by qPCR. The gene expression levels of Ccnd1, Fn1 and Tgfb1 were low in aortae from both Ctrl and CRF rats. No differences were seen in the aorta gene expression of Ctnnb1, Ccnd1, Jun, Snai1, Vim, Fn1, Postn, Inhba and Tgfb1 between Ctrl and CRF rats. The alfacalcidol-treated CRF-D rats had significantly increased aorta gene expression of Ctnnb1, Ccnd1, Tgfb1, Snai1, Vim, Fn1, Inhba and Postn compared to Ctrl (P<0.05), whereas Jun expression was unaffected.

ICG-001-administered Ctrl rats had significantly increased aorta gene expression of Jun compared to vehicle-administered Ctrl (P<0.05). No other differences were seen in gene expression between aortae from ICG-001- and vehicle-administered Ctrl. Likewise, no differences in aorta gene expression were seen between ICG-001- and vehicle-administered CRF and CRF-D rats.

### Effects of ICG-001 on protein expression and vascular calcification in the uremic aorta and in the aorta from control rats

**Fig 5:**
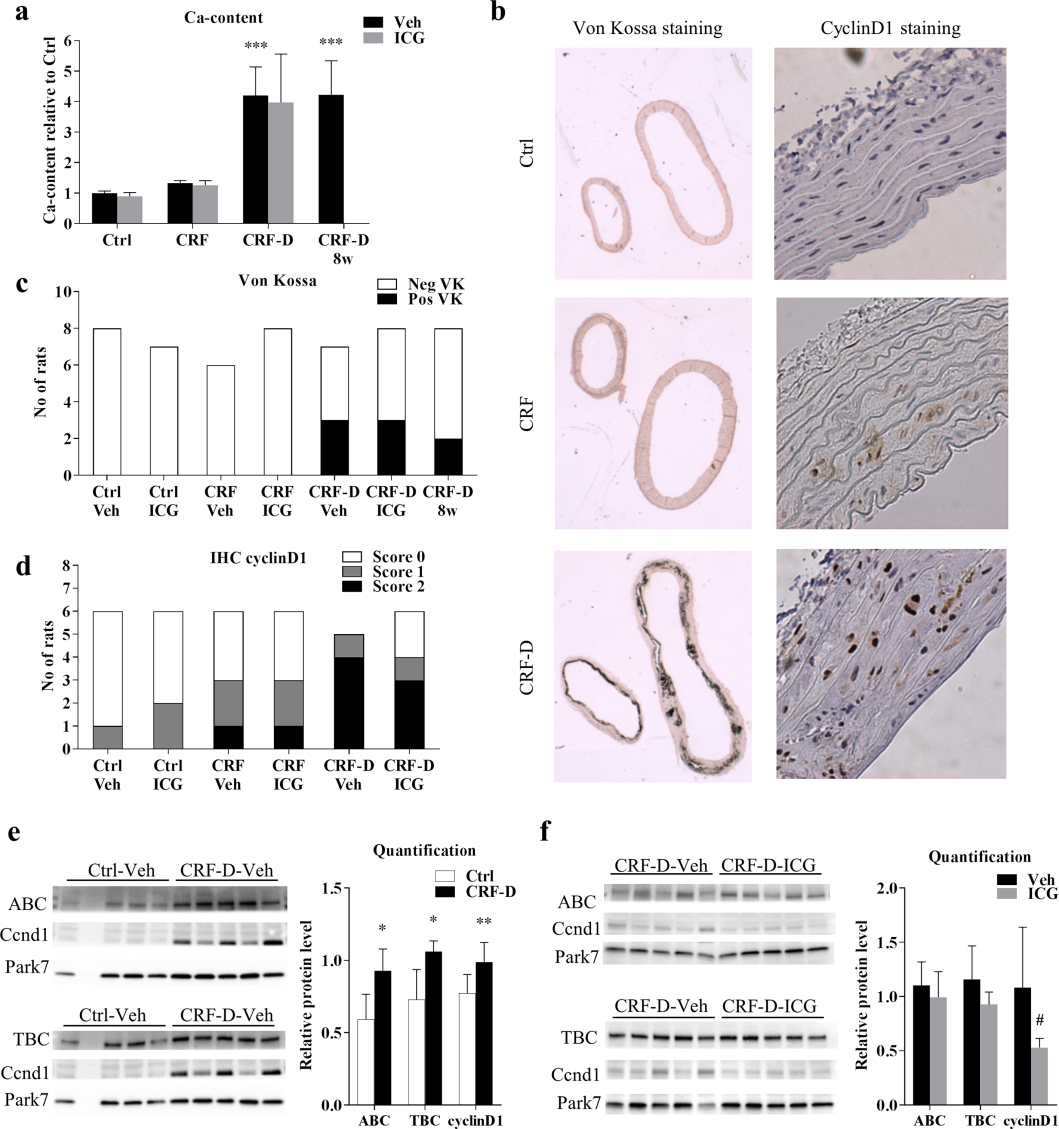
Aorta calcification and protein expression. Ca-content was determined in the proximal thoracic aorta. A marked increase in Ca-content was seen in CRF-D rats, while no difference was seen between CRF and Ctrl. No differences were seen in Ca-content between ICG-001- and vehicle-administered rats (**a**). Representative pictures of von Kossa and cyclinD1 stains are presented in (**b**). No Ctrl or CRF rats stained positive by VK. Three rats stained positive in both the ICG-001- and vehicle-administered CRF-D groups (**c**), and noticeably all rats staining positive by VK had massive, confluent staining, both in the proximal thoracic (right side of the image) and distal abdominal aorta (left side of the image). Aortic protein expression of cyclinD1 was examined by IHC (**d**). The majority of Ctrl rats stained negative and approximately half of the CRF rats stained positive for cyclinD1. No differences were noted between ICG-001- and vehicle-administered Ctrl and CRF rats. All vehicle-administered CRF-D rats stained positive for cyclinD1, while two ICG-001-administered CRF-D rats stained negative. CyclinD1, total β-catenin (TBC) and active β-catenin (ABC) protein levels were examined by WB in aortae from vehicle-administered Ctrl and CRF-D rats (**e**) and in aortae from ICG-001 and vehicle-administered CRF-D rats (**f**). Aortic protein levels of cyclinD1, total and active β-catenin were increased in vehicle-administered CRF-D rats compared to Ctrl, and protein levels of cyclinD1 were reduced in ICG-001-admininstered CRF-D rats compared to vehicle-administered CRF-D rats. Blots cropped from different parts of the same gel are presented together. Uncropped blots are provided in S1 Fig. Data is presented as mean ± SD. n = 5–9. Vehicle-treated Ctrl, CRF and CRF-D rats were compared by one-way ANOVA and Dunnets multiple comparison with ***P<0.001 vs Ctrl. ICG-001- and vehicle-administered groups were compared using unpaired two-tailed t-test with ^#^P <0.05 vs vehicle. In WB (**e**) Ctrl and CRF-D rats were compared by unpaired two-tailed t-test with *P<0.05 and **P<0.001 vs Ctrl.

VC in the proximal thoracic aorta was examined by von Kossa staining and the total Ca-content was determined. Ca-content of the proximal thoracic aorta was markedly increased in CRF-D rats compared to Ctrl rats (P = 0.0001), whereas no significant difference was seen in Ca-content between aorta from CRF and Ctrl rats (Fig 5A). No differences in Ca-content between ICG-001- and vehicle-administered rats were noted. A subgroup of CRF-D rats had manifest calcifications on the von Kossa staining (Fig 5B and 5C). The protein expression of cyclinD1 was examined by immunohistochemistry (IHC), and positive cyclinD1 staining was observed in the nucleus of the vascular smooth muscle cells (VSMC). CyclinD1 staining was negative in the majority of Ctrl rats, although weak staining was noted in a few rats. A positive cyclinD1 staining was however seen in 50% of the CRF rats, but with no difference between ICG-001- and vehicle-administered rats. All vehicle-administered CRF-D rats stained positive, whereas in the ICG-001-administered CRF-D group two out of six rats stained negative, while four out of six rats stained positive (Fig 5D). As the IHC-staining only provided a semi-quantitative measure, aorta protein expression of cyclinD1 was further quantified by Western blot. Thus, the protein levels of cyclinD1, as well as total and active β-catenin were examined by Western blot in aorta from vehicle-administered Ctrl rats and from ICG-001- and vehicle-administered CRF-D rats. In agreement with the IHC result, a significant increase in cyclinD1 protein levels was seen in aorta from CRF-D rats compared to Ctrl (P<0.01), and significant increases in protein levels of both total and active β-catenin were seen in aortae from CRF-D rats compared to Ctrl (P<0.05) (Fig 5E). ICG-001-administered CRF-D rats had unchanged protein levels of total and active β-catenin compared to vehicle treated CRF-D rats (Fig 5F). ICG-001 administration in CRF-D rats resulted in significantly lower cyclinD1 protein levels compared to vehicle-administered CRF-D rats (P<0.05) (Fig 4F).

### Characterization of the disturbed gene expression in the uremic calcified aorta

**Fig 6:**
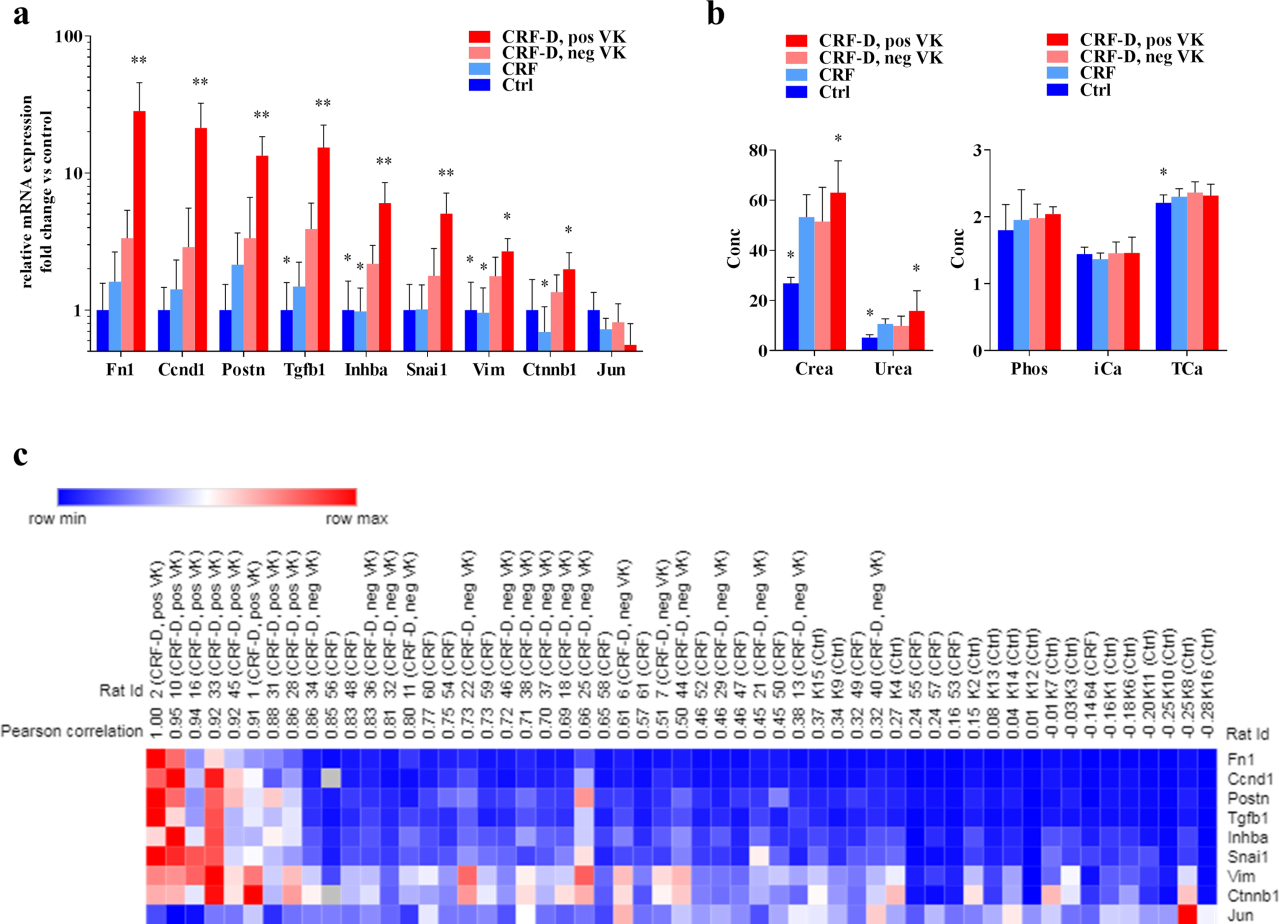
VC-associated disturbances in aorta gene expression. Gene expression in aortae with manifest calcifications by VK stain were compared with aortae from CRF-D rats with negative VK stain and further gene expression in aortae from CRF and Ctrl rats were compared to CRF-D rats with negative VK (**a**). The presence of manifest calcifications was associated with a massive shift in gene expression, which was dampened in aortae from CFR-D rats with negative VK. The rats with positive VK were further characterized by increased plasma levels of creatinine and urea (**b**), while no differences in plasma levels of phosphate, Ca^2+^ and TCa were noted between VK positive and VK negative CRF-D rats (**b**). A heatmap was generated, where rats were ranked according to the Pearson correlation coefficient and compared to the rat with the highest expression of Fn1 (ID = 2) (**c**). In the heatmap the rats with positive VK stain were grouped together and Ctrl rats were located in the opposite end, while the CRF-D rats with negative VK and CRF rats were found in the middle, and were not readily separated. Data is presented as mean ± SD. n = 8–17. Groups were compared by one-way ANOVA and Dunnets multiple comparison with *P<0.05 and **P = 0.0001 vs CRF-D with neg. VK.

To further examine the disturbed gene expression in the calcified aorta, CRF-D rats with positive VK stain was compared to CRF-D rats with negative VK stain and further compared to CRF and Ctrl. As no differences were noted in aorta gene expression between ICG-001- and vehicle-administered rats, ICG-001 and vehicle groups were pooled. The presence of manifest calcifications as defined by positive VK stain was accompanied by an enormous shift in gene expression (P<0.0001), which was blunted in the CRF-D rats with negative VK stain (Fig 6A). The genes with the highest relative increases (>5-fold vs Ctrl) were Fn1, Ccnd1, Postn, Tgfb1 and Inhba. The rats with positive VK stain were further characterized by higher plasma levels of creatinine and urea (P<0.05), whereas no differences in plasma phosphate, Ca^2+^ or total Ca were seen between the groups (Fig 6B). A heatmap of the aorta gene expression was created. Aorta gene expression was correlated to the rat with the highest expression of fibronectin (Fn1) (rat ID = 2), and ranked according to the Pearson correlation coefficient. The heatmap demonstrates that a subset of the examined genes was capable of distinguishing the rats with positive VK stain from the remainder of the rats. The Ctrl rats were grouped opposed to the CRF-D rats with positive VK stain, and strikingly, the CRF-D rats with negative VK stain were not readily separated from the CRF rats in the heatmap (Fig 6C).

### Effects of ICG-001 on bone morphology and bone gene expression

**Fig 7:**
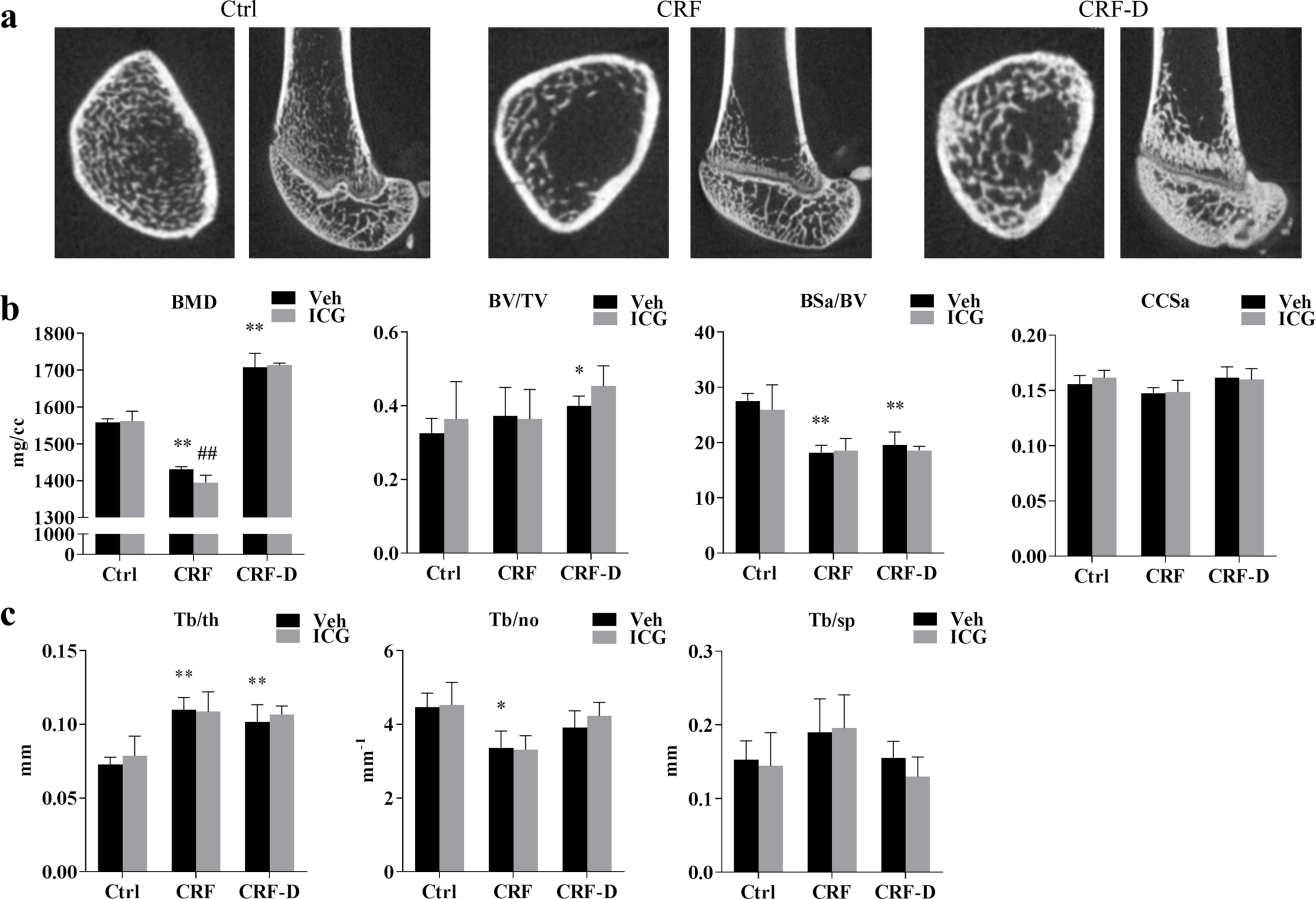
Bone μCT data. Bone microstructure was examined by μCT scan. Representative pictures are presented in (**a**). Trabecular bone mineral density (BMD), bone volume/total volume (BV/TV), bone surface area/BV (BSa/BV) and cortical cross-sectional area (CCSa) is presented in (**b**). Trabecular thickness (Tb/th), number (Tb/no) and spacing (Tb/sp) is presented in (**c**). Trabecular BMD was decreased in CRF rats while the trabecular BMD and bone volume were increased in CRF-D rats. The ICG-001 administration resulted in a further decrease in trabecular BMD in CRF rats. In both CRF and CRF-D rats bone surface area was reduced and trabecular thickness was increased compared to Ctrl. Trabecular number was reduced in CRF rats and a trend towards an increase in trabecular spacing was seen in CRF, and these changes were reduced in CRF-D rats. Data is presented as mean ± SD. n = 3–7. Vehicle-administered Ctrl, CRF and CRF-D rats were compared by one-way ANOVA and Dunnets multiple comparison with *P<0.05 and **P<0.001 vs Ctrl. ICG-001- and vehicle-administered groups were compared using unpaired two-tailed t-test with ^##^P <0.01 vs vehicle.

As Wnt signalling is active in adult bone, the potential off-target effects of ICG-001 on bone morphology, trabecular bone mineral density (BMD) and gene expression were examined by microCT (μCT) and qPCR. Representative μCT pictures are presented in Fig 7A. CRF rats had decreased trabecular BMD (P<0.001) and unchanged bone volume fraction (BV/TV), compared to Ctrl, whereas an increase in trabecular BMD and bone volume fraction was seen CRF-D rats (P<0.001), compared to Ctrl. Bone surface area (BSa/BV) was decreased in both CRF and CRF-D rats (P<0.001) compared to Ctrl and no differences were seen between groups in cortical cross-sectional area (CCSa) (Fig 7B). Trabecular thickness (Tb/th) was increased both in CRF and CRF-D rats compared to Ctrl (P<0.001), whereas trabecular number (Tb/no) was decreased in CRF (P<0.05) and not significantly different in CRF-D rats compared to Ctrl (Fig 7C). No significant differences were seen in trabecular spacing (Tb/sp) between groups (Fig 7C). ICG-001-administered CRF rats had significantly lower trabecular BMD compared to vehicle-administered CRF rats (P<0.01), while no differences in trabecular BMD were seen between ICG-001- and vehicle-administered Ctrl or CRF-D rats. Furthermore, no differences between ICG-001- and vehicle-administered rats were found in the bone volume fraction, bone surface area, cortical cross-sectional area or in trabecular thickness, number or spacing.

mRNA levels of the early osteoblast marker runt-related transcription factor (Runx2), the mature osteocyte marker sclerostin (Sost) and the osteoclast differentiation factor rank ligand (Tnfsf11), as well as mRNA levels of β-catenin (Ctnbb1), cyclinD1 (Ccnd1) and Jun were determined in femoral cortical bone tissue. The data are presented in S3 Fig. mRNA-levels of Runx2 and Sost were decreased in CRF rats compared to Ctrl (P<0.05). Likewise, Runx2 mRNA levels were decreased in CRF-D rats compared to Ctrl (P<0.05). A significant increase in Rankl mRNA levels was seen in CRF-D rats compared to Ctrl (P<0.05). No significant differences were seen in mRNA levels of Ctnbb1, Ccnd1 and Jun between Ctrl rats and CRF or CRF-D rats. ICG-001-administered CRF-D rats had significantly decreased levels of Rankl (P<0.05), increased levels of β-catenin (P<0.05) and decreased levels of Jun mRNA (P<0.01) compared to vehicle-administered CRF-D rats. No significant differences were found in mRNA levels between ICG-001- or vehicle-administered Ctrl and CRF rats.

## Discussion

The involvement of Wnt signalling and the effect of Wnt inhibition on established uremic vascular calcification were examined in the present study. The small molecule compound ICG-001 was used to inhibit Wnt signalling. ICG-001 specifically targets the β-catenin binding site on CBP, and thereby hinders CBP-activated β-catenin signalling [21]. Vascular calcification was induced by long term uremia, high phosphate diet and alfacalcidol treatment in 5/6-nephrectomized rats. The Wnt pathway was significantly disturbed in the uremic, calcified aorta. Treatment with ICG-001 ameliorated the expression of cyclinD1. Surprisingly, administration of ICG-001 did not reverse the established vascular calcifications.

To examine the bioavailability and the antifibrotic effects of ICG-001, a short-term experiment was first conducted in the unilateral ureteral obstruction (UUO) model. Renal fibrosis is the final common pathway of all progressive renal diseases [23]. Several studies and previous results from our group have shown that kidney injury could reactivate developmental programs involved in nephrogenesis, attempting renal repair [24–27]. Wnt family members and factors controlling Wnt function are critical morphogens in the developing kidney [28]. Although Wnt signalling have been implied in the regeneration of injured of kidney tissue, persistent activation and dysregulation of the Wnt pathways underlie fibrosis and progressive renal failure [28]. In agreement with previously reported results from our group, UUO was associated with induction of expression of genes related to fibrosis [27]. ICG-001 abolished the induced expression of pro-fibrotic genes in the UUO experiment, confirming the bioavailability and effect of the compound, in agreement with previous results by Hao et al [19]. The UUO model is an experimental model of unilateral progressive renal fibrosis in the obstructed kidney, which takes place in a non-uremic environment due to the functioning untouched contralateral kidney [29]. In the 5/6-nephrectomy model, the remnant kidney is morphologically characterized by progressive glomerulosclerosis and tubulointerstitial fibrosis[30], but in contrast to the UUO model, this takes place in an uremic milieu. ICG-001 could potentially have anti-fibrotic effects on the remnant kidney in the 5/6-nephrectomy model and hereby improve kidney function, and could therefore theoretically both have direct and indirect beneficial effects on the vasculature. Therefore, the effects of ICG-001 on kidney gene and protein expression were examined in CRF rats and in Ctrl rats from our vascular calcification study. In the kidneys from Ctrl rats ICG-001 resulted in a decrease in the gene expression of snail 1 and vimentin, as well as in a decrease in the gene expression of TGF-β, β-catenin and cyclinD1 compared to vehicle-administered Ctrl. These results are in agreement with the significant effects seen in the UUO model in the present study. The ICG-001-induced decrease in cyclinD1 and β-catenin gene expression in kidneys from Ctrl rats was confirmed at the protein level by Western blot. No effects of ICG-001 were however found on either gene or protein expression in kidneys from CRF rats, despite the significant effects of ICG-001 on kidney gene expression in both the UUO model and in Ctrl rats from the vascular calcification model.

The reason for this lack of effect of ICG-001 on the fibrotic remnant uremic kidney remains uncertain. Even though the genes related to fibrosis were induced in kidneys from CRF rats, β-catenin gene expression was similar to that of Ctrl rats, and cyclinD1 gene expression was suppressed compared to Ctrl rats. The suppressed cyclinD1 levels might be due to cell cycle arrest in the established fibrosis, as previously described [31, 32]. Furthermore, even though the ICG-001 had an antifibrotic effect when given as a preventive treatment in the UUO model, the ICG-001 was administered at a time when established kidney fibrosis was already present in the vascular calcification model. Thus, potentially Wnt signalling was involved in the development of kidney fibrosis, but selective targeting the CBP/β-catenin-mediated Wnt signalling was insufficient to amend the established fibrotic changes. Furthermore, uremia by itself might modify the effects of ICG-001. In this context it should be noted that the plasma levels of the circulating Wnt inhibitors sclerosin (Sost) and dickkopf-related protein 1 (Dkk1) are increased in uremia [33] and advanced uremia might per se be associated with a general state of Wnt inhibition, which potentially could make pharmacological inhibition of Wnt signalling less efficient.

Several studies have identified Wnt signalling as being a key player in the development of vascular pathology [14]. This emphasizes the relevance of therapeutic strategies targeting components of the Wnt signalling pathway in the treatment of uremic vascular calcification. VSMC in the vascular lamina media normally exhibit a low proliferation rate [34]. Uremia results in the activation and dedifferentiation of VSMC toward a synthetic, osteochondrogenic phenotype with the capability to proliferate, migrate and increase the production of extracellular matrix [6]. The effect of β-catenin on VSMC proliferation is well-established [14], and β-Catenin/TCF signalling can upregulate the expression of proliferative genes such as cyclinD1 [17]. In the present model of uremic vascular calcification, the CRF-D rats had increased aorta Ca-content, and a subgroup of CRF-D rats stained positive by von Kossa as a sign of a more advanced calcification process. Aorta gene and protein expression of β-catenin and cyclinD1, a reported target of CBP-coactivated Wnt signalling, were induced in the CRF-D rats, while the expression of the Jun gene, a reported target of p300-coactivated Wnt signalling, was unchanged. These results are in agreement with those of our previous RNAseq study [15], and might suggest an activation of CBP-coactivated β-catenin-mediated Wnt signalling in the aorta from these rats. In Ctrl rats ICG-001 administration resulted in an induction of Jun gene expression, which is consistent with a shift towards p300-coactivated signalling [18]. As VSMC are quiescent in healthy vasculature [34], cyclinD1 gene and protein expression were very low in aorta from Ctrl rats and there was no detectable effect of ICG-001 on cyclinD1 in Ctrl aortas. In aortae from CRF-D rats no effects of ICG-001 were seen on gene expression of Wnt-related genes and markers of fibrosis, EMT and matricellular proteins. This finding is surprising as fibronectin, periostin and snail1 are reported to be targets of Wnt signalling [35–37], and further surprising as we previously have shown that the pro-fibrotic gene expression induced in the uremic, calcified aorta in the present model is modifiable, and does respond to treatment with the TGF-β antagonist, BMP7 [38]. In our previous study BMP7 treatment resulted in a reduction in the expression of fibronectin, periostin, inhibin-βa and snail1, while the expression of these genes was unaffected by ICG-001 treatment in the present investigation. Although TGF-β and Wnt signalling are closely intertwined [39], the examined genes were apparently more receptive to modulation of the TGF-β signalling pathway.

Although cyclinD1 gene expression was similar in the aorta from ICG-001- and vehicle-administered CRF-D rats, a number of the ICG-001-administered CRF-D rats stained negative for aorta cyclinD1 by immunohistochemistry staining. Therefore, the protein levels of cyclinD1 were quantified by Western blot, which demonstrated a significant reduction in protein levels of cyclinD1 in aortae from ICG-001 treated CRF-D rats compared to vehicle-administered CRF-D rats. ICG-001 did not reduce expression of Wnt-related genes and markers of fibrosis, EMT and matricellular proteins in CRF-D rats except for cyclinD1. These results might indicate that other signalling pathways such as TGF-β and activin could be involved in the transcriptional alterations induced in the present model of advanced vascular calcification. Furthermore, research from several groups indicates a possible role of non-canonical Wnt signalling in atherosclerosis [14], and non-canonical Wnt signalling might potentially also be involved in uremic vascular calcification. Noticeably, the most upregulated Wnt ligand in the vasculature was Wnt16 (please, see S1 Table), that has been shown to induce both non-canonical and canonical Wnt signalling [40]. Although our data suggest that there is activation of canonical Wnt signalling in uremic vascular calcification, the data does not exclude a concurrent induction of non-canonical Wnt signalling with potential pro-calcific effects.

ICG-001 administration significantly increased the expression of jun in aortae from Ctrl rats, while there was no effect in uremic rats. This is in parallel with the effects of ICG-001 on kidneys from Ctrl rats, and with no effect on kidneys from CRF rats. As noted above, uremia is associated with increased circulating levels of Wnt inhibitors, which could modify the effects of ICG-001. It should also be noted, that the present model of uremic vascular calcification is associated with an increased expression of the Wnt inhibitor sclerostin in the aorta (S1 Table). This might imply the presence of local endogenous Wnt inhibitors in the vasculature. Sclerostin is primarily expressed in osteocytes and chondrocytes and it inhibits bone formation by osteoblasts. Thus, the increased levels of sclerostin in the calcified aorta might have a protective effect against osteochondrogenic transformation in the lamina media. The precise role of sclerostin in the vasculature has however not yet been established. Interestingly, the increased circulating levels of the Wnt inhibitors sclerostin and Dkk1 have in uremia been suggested to be involved in the pathogenesis of the CKD-MBD [33], and a role of neutralizing antibodies against circulating Wnt-inhibitors has been proposed for treatment of CKD-MBD [41].

The disturbed gene expression associated with the presence of severe calcifications in aortae from von Kossa positive CRF-D rats was compared with gene expression in aortae from von Kossa negative CRF-D rats. The presence of von Kossa positive calcifications was associated with a massive induction of the examined genes; fibronectin, cyclinD1, periostin, TGF-β, inhibin-βa, snail1, vimentin and β-catenin, except for Jun, and this induction was blunted in the CRF-D rats with negative von Kossa staining. This finding might suggest that the incorporation of calcium and phosphorus deposits in the vasculature is associated with changes in the VSMC phenotype that at some point intensifies or accelerates. This notion is further supported by the fact, that all rats with positive von Kossa had massive calcifications throughout most of the medial layer, and no sporadic calcifications were seen. Noticeably, the rats with positive von Kossa stain were slightly more uremic compared to von Kossa negative rats, whereas no differences in plasma calcium and phosphate were noted.

The main purpose of the present study was to investigate the reversibility of uremic vascular calcification by treatment with ICG-001. No effect of ICG-001 was seen on the Ca-content of the aorta, and a comparable number of rats stained positive by von Kossa in ICG-001- and vehicle-administered CRF-D rats. Even though we previously have shown, that the profibrotic gene expression in the calcified aorta can be ameliorated by treatment with BMP7, the established vascular calcification was not reversible by the BMP7 treatment or by the ICG-001 used in the present investigation. These findings indicate that the accumulation of calcium and phosphorus in the vascular wall is not readily reversible and will potentially require the presence of specialized cells able to digest the calcium and phosphate crystals [38].

Wnt signalling is active in adult bone; consequently, caution should be taken with regard to bone health when systemically administering a Wnt inhibitor. Therefore, the potential effects on bone microstructure were examined by μCT and a negative impact of ICG-001 on bone in CRF rats was revealed. CRF rats had decreased trabecular BMD compared to Ctrl rats, and the ICG-001 resulted in a further reduction in BMD in CRF rats probably due to further inhibition of Wnt signalling in bone, in addition to the well-known resistance to PTH [42]. ICG-001 did not reduce BMD in the heavily calcified, adynamic bone from CRF-D rats. Of importance, ICG-001 had no impact on bone microstructure in normal control rats. This potential functional redundancy and difference in effects between the coactivators CBP and p300 in the regulation of Wnt signalling in bone has not previously been described. Although much focus has been drawn to CBP and p300 a number of other β-catenin coactivators exist [43], which potentially could be recruited when the CBP/β-catenin interaction is blocked. By specifically targeting the CBP/β-catenin interaction, it is possible that the risk of potential off-targets effects on normal bone was reduced.

CBP/β-catenin antagonists are currently used in preclinical and clinical investigations as treatment for advanced solid tumors and advanced myeloid malignancies. The use of ICG-001 in clinical studies creates a huge significant requirement not only for knowing the effects, but also for studying the potential side effects of this compound. The results of the present study, which show a clear effect of ICG-001 on bone, but also on normal vasculature and kidneys should be considered in this context. The limited effect of ICG-001 on the calcified vasculature and the lack of effect on long term kidney fibrosis in uremia are surprising. In the present investigation a rather high dose of the compound was used, which previously has been shown to be effective in different models of organ fibrosis [19, 20, 44, 45]. The effect of increasing doses of ICG-001 will be subject for future experimental studies.

In conclusion, the presence of uremic vascular calcification is associated with a massive shift in aorta gene expression, and in induction of the expression of Wnt-related genes and proteins, including β-catenin and cyclinD1. Although Wnt inhibition by ICG-001 had significant effects on protein and gene expression in kidneys and aortae from normal control rats, these effects were limited in uremia, and ICG-001 treatment did not reduce the Ca-content of the uremic aorta. The present results indicate induction of Wnt signalling in the calcified aorta, although the specific inhibition of CBP-coactivated β-catenin signalling did not reduce the degree of vascular calcification. Therefore, the lack of reversibility of aorta calcification in the current study stresses the importance of preventing the development of vascular calcification in chronic kidney disease.

## Materials and methods

### Animals and experimental models

Inbred adult male Dark Agouti (DA) rats weighing 200g (Envigo, The Netherlands) were used in the vascular calcification study. Rats were housed in a temperature controlled environment with a 12-hour light/dark cycle and *ad libitum* access to water and diet. Chronic renal failure (CRF) was induced by 5/6-nephrectomy. The 5/6-nephrectomy was performed as a one-step procedure through an incision in the back, as previously described [46]. Rats were anaesthetized with Hypnorm/Midazolam (2μL/g; Panum Institute, Copenhagen, Denmark). Carprofen (Pfizer, Denmark) was given subcutaneously as pain relief for three days. CRF rats were fed a high phosphate diet throughout the study (0.9%Ca, 1.2%P, 600IU vitamin D per kg diet; Altromin Spezialfutter GmbH & Co, Germany) and control rats were fed standard diet (0.9% Ca, 0.7% P, 600IU vitamin D per kg diet; Altromin). To increase the development of VC, CRF rats were administered alfacalcidol (Leo Pharmaceuticals, Denmark) 80 ng intraperitoneally (ip) three times weekly for four weeks. In the UUO study adult male Wistar rats (Taconic A/S, Denmark) were used. Briefly, in the UUO rats the left ureter was ligated under general anaesthesia and the ligature was kept until sacrifice after 72 hours. ICG-001 (Cat#4505, Tocris, Bio-Techne, UK) was administered daily as ip injections at a dose of 5mg/kg, equivalent to the dosing used in previous studies [19, 44, 45, 47, 48]. ICG-001 was dissolved in 96% ethanol and the injected volume was 50μL/day. Vehicle consisted of 50μL 96% ethanol.

### Ethics

The experimental studies were approved by the Animal Experiments Inspectorate, Denmark (Reference: 2017-15-0201-01214) and performed in accordance with the NIH Guide for the Care and Use of Laboratory Animals. The rats were under daily supervision by the researchers and the animal caretakers from Department of Experimental Medicine, The Panum Institute, University of Copenhagen, and animals were sacrificed if failure to thrive was noted.

### Experimental design

The UUO study was performed in order to examine the effect and bioavailability of the commercially available ICG-001. In a short-term experiment the effect of ICG-001 on kidney gene expression was examined in the unilateral ureteral obstruction model (UUO model), where the contralateral kidney is left untouched. Rats were allocated to Ctrl, UUO/Veh or UUO/ICG. ICG-001 or vehicle was administered at the time of surgery and daily for three days with the last dose given in the morning on the day of sacrifice.

The vascular calcification study examined the effect of four weeks treatment with ICG-001 on established VC in chronic uremia. ICG-001 was administered after induction of VC in CRF rats with high phosphate diet and alfacalcidol (CRF-D). A control group of uremic rats not treated with alfalcalcidol (CRF) was used to examine the potential effects of Wnt inhibition on uremia and on the fibrotic remnant kidney of the 5/6-nephrectomy model. After four weeks of uremia CRF rats were allocated to five experimental groups according to weight and plasma urea as outlined in Fig 1. A group of normal age-matched rats (Ctrl) were kept in parallel and allocated to either ICG-001 or vehicle. The last ICG-001 or vehicle injection was administered in the morning on the day of sacrifice. At sacrifice rats were anaesthetized with pentobarbital (50μg/kg ip; Nycomed-DAK, Denmark) and eye-blood was drawn. The aorta was dissected, removing blood and connective tissue, and kidney rudiments from CRF rats and corresponding segments from Ctrl rats were collected. Cortical bone from the right femur was harvested for qPCR and the left femur was placed in 70% ethanol and stored at 5°C for μCT scan.

### Plasma biochemistry and aorta Ca-content

Plasma creatinine, urea, phosphate and total Ca were analyzed by Vitros 250 (Ortho-Clinical Diagnostics, Raritan, USA). Plasma Ca^2+^ was measured by ABL800 (Radiometer, Copenhagen, Denmark). Plasma intact FGF23 (iFGF23) was measured by a human FGF23 ELISA (Kainos Laboratories, Tokyo, Japan), with an intra-assay coefficient of variation of 2.5% and inter-assay coefficient of variation of 5% in our lab [49]. Plasma PTH was measured by a rat bioactive intact PTH ELISA assay (Immutopics, San Clemente, USA) with an intra-assay coefficient of variation of 4% and intra-assay variation of 9% in our lab [50]. Aorta Ca-content was determined by the o-cresolphthalein method and normalized to the dry weight. A small section of the proximal thoracic aorta was lyophilized for 24 hours to determine the dry weight. After lyophilisation the aorta section was decalcified in 1M HCl for 24 hours and the Ca-content of the supernatant was determined using a commercial assay (Sigma-Aldrich, St. Louis, USA).

### Quantitative RT-PCR

Thoracic aorta, cortical bone and kidney tissue were manually ground by mortar and pestle immersed in liquid nitrogen. Total RNA was extracted from the tissue-powder using the EZNA RNA kit (Omega Bio-tek, GA, USA). First strand cDNA was synthesized from 0.2–1.5μg of RNA with Superscript III cDNA kit (Invitrogen, MA, USA). Jumpstart (Sigma-Aldrich, MO, USA) and Lightcycler 480II (Roche, Basel, Switzerland) were used for qRT-PCR, with a binding temperature of 59°C. The mRNA levels were normalized to the mean of reference genes Arbp and Rpl13a and reference gene stability was confirmed using Genorm [51]. Primers are listed in S3 Table.

### Western blot

Protein was extracted from kidney and thoracic aorta in T-PER (Thermo Scientific, Rockford, IL) with Halt protease and phosphatase inhibitor cocktail (Thermo Scientific). The protein concentration was determined by the BCA assay (Thermo Scientific). 30 μg of protein was run on Mini-Protean precast gel (Bio-Rad, Munich, Germany) and transferred onto nitrocellulose membranes (Bio-Rad). Membranes were blocked with 5% bovine serum albumin (BSA) (Roche, Mannheim, Germany) in PBS with 0.05% tween. Primary and secondary antibodies were diluted in PBS with 3% BSA. The antibodies used were monoclonal anti-human cyclinD1 (1:500, Cat#2978, Cell Signaling Technology), monoclonal anti-mouse total β-catenin (1:2000, Cat#610153, BD Biosciences), monoclonal active β-catenin (1:500, Cat#05–665, Merck Millipore), polyclonal anti-human park7 (1:1000000, Cat#ab18257, Abcam). The active β-catenin antibody specifically detects β-catenin dephosphorylated at Ser37 and Thr41, as this state of β-catenin has been shown to mediate Wnt-signalling [52]. The total β-catenin antibody binds the C-terminal region of β-catenin. The secondary antibodies used were HRP-conjugated anti-rabbit (1:2000, Cat#P0448, Dako) and anti-mouse (1:1000, Cat#P0447, Dako). Blots were visualised by the Amersham ECL Prime Detection Reagent (GE Healthcare, Freiburg, Germany) using the Chemidoc XRS+ System (Bio-Rad). Western blot quantifications were performed with ImageJ.

### Histology and immunohistochemistry

For von Kossa (VK) and immunohistochemistry (IHC) staining, sections of the proximal thoracic aorta and distal abdominal aorta were fixed in 10% buffered formalin for 24–48 hours at room temperature. After formalin fixation tissue sections were dehydrated and embedded into paraffin blocks. 4μm sections were cut and stored at -20°C until staining. VK staining was performed according to standard protocols. IHC staining was performed using the Dako Link Autostainer in an automated protocol. Deparaffinization, rehydration and heat-induced antigen retrieval was performed in EnVision Flex Target Retrieval Solution at pH = 9 in the PT Link module (Agilent Dako, Copenhagen, Denmark). Endogenous peroxidase activity was blocked by incubation with EnVision FLEX Peroxidase-Blocking Reagent (Agilent Dako). Sections were incubated with rabbit anti-human cyclinD1 antibody (Clone EP12, Agilent Dako), followed by HRP-conjugated secondary anti-rabbit antibody (Agilent Dako) and Flex DAB Chromogen (Agilent Dako). Slides were counterstained with hematoxylin. Background staining was determined in sections incubated without primary antibody. Images were acquired on Axio Imager Z1 with Axiocam (Carl Zeiss, Germany). All specimens were blinded before scoring. CyclinD1-stained sections were quantified on a scale from 0–2 with 0 = negative, 1 = low positive, 2 = high positive. VK-stained sections were scored on a scale from 1–6, as previously described [15], but as almost all sections either scored 1 (negative) or 6 (high), results are reported as positive or negative.

### Micro-computed tomography (μCT)

High resolution micro-computed tomography (μCT) was performed on the whole femur. CT images were acquired on an Inveon Multimodality PET/CT scanner (Siemens, USA) with the following settings: 361 projections, 60 kV, 500 μA and 1300 ms exposure. Images were reconstructed with an isotropic voxel size of 32 μm. Image analysis was performed using the Inveon Software (Siemens). The distal femur growth plate was used as reference. The cortical bone cross section area (CCSa) was measured mid shaft (10 mm proximal to the reference growth plate) by manually drawing the outer and inner perimeter, and then subtracting the ellipsoid areas. For analysis of trabecular bone, a region of interest (ROI) of 0.960 mm along the longitudinal direction was drawn manually starting at 2.080 mm proximal to the reference growth plate. The CT images of ROI were segmented into bone and marrow by a visually chosen fixed threshold for all groups. Bone morphometry was calculated using the Inveon Software based on the parallel plate model by Parfitt [53]. The following parameters were calculated: the ratio of total trabecular volume to total tissue volume (BV/TV), the ratio of trabecular bone surface to trabecular bone volume (BSa/BV), trabecular thickness (Tb/th), trabecular number (Tb/no), trabecular spacing (Tb/sp). A standard bone phantom (Inveon, Siemens) was calibrated and applied to calculate the BMD of the trabecular bone.

### Statistics

Data are presented as mean ± standard deviation (SD). One-way analysis of variance (ANOVA) and Dunnet’s post-hoc test was used to test for between-group differences in mean. Two-tailed unpaired t-test was used to compare means between ICG-001- and vehicle-administered groups. P<0.05 was considered significant. Calculations were performed in Graphpad Prism 7.0. Heatmap was generated using the publically available Morpheus Software provided by Broad Institute (Cambridge, MA) (https://software.broadinstitute.org/morpheus/).

## Supporting information

S1 Table Aortic expression of genes related to the Wnt-signaling pathway. In a previous study we performed RNA deep sequencing of aortae from Ctrl rats and uremic rats with vascular calcification [15]. The database was searched for genes related to Wnt-signaling; specifically, Wnt ligands, intracellular Wnt-signaling transducers, Wnt target genes and Wnt inhibitors were searched. Only genes with significant differences between uremic and Ctrl rats are presented.
(PDF)

S2 Table Plasma biochemistry and body weight at sacrifice. Data is presented as mean ± SD and PTH as median and [range]. n = 6–9. Vehicle-treated Ctrl, CRF and CRF-D rats were compared by one-way ANOVA and Dunnets multiple comparison with *P<0.05, **P<0.001 and ***P<0.0001 vs Ctrl. ICG-001- and vehicle-administered groups were compared using unpaired two-tailed t-test with ^#^P <0.05 vs vehicle.
(PDF)

S3 Table Primer sequences. (PDF)

S1 Fig Effect of ICG-001 on kidney gene expression in unilateral ureteral obstruction (UUO). ICG-001 was administered at the time of obstruction and subsequently daily at a dose of 5mg/kg. Rats were sacrificed after three days of UUO. Kidney gene expression was examined in the obstructed kidney from ICG-001 (UUO-ICG) and vehicle treated rats (UUO-Veh) as well as in the normal kidney from untouched control rats (Ctrl). UUO resulted in an induction in kidney expression of profibrotic genes, and ICG-001 administration ameliorated this response. Data is presented as mean ± SD. Data is presented as mean ± SD. n = 6–9. *P<0.05 vs Ctrl by unpaired two-tailed t-test, ^#^P <0.05 vs vehicle by unpaired two-tailed t-test.
(TIF)

S2 Fig Uncropped WB. (**a**) WB from Fig 3B, (**b**) WB from Fig 3D, (**c**) WB from Fig 4E, (**d**) WB from Fig 4F.
(TIF)

S3 Fig Bone gene expression. Gene expression was examined in cortical femoral bone tissue by qPCR. Gene expression of the early osteoblast marker Runx2 and the mature osteocyte marker Sost was decreased in CRF and CRF-D rats compared to Ctrl, whereas the osteoclast differentiation marker Rankl was increased in CRF-D rats compared to Ctrl. ICG-001 treatment resulted in a decrease in the expression of Rankl in CRF-D rats, and surprisingly an increase in Ctnnb1 and a decrease in Jun was seen in ICG-001 treated CRF-D rats compared to vehicle-treated CRF-D rats. Data is presented as mean ± SD. n = 6–9. Vehicle-treated Ctrl, CRF and CRF-D rats were compared by one-way ANOVA and Dunnets multiple comparison with *P<0.05 and **P<0.001 vs Ctrl. ICG-001- and vehicle-treated groups were compared using unpaired two-tailed t-test with ^#^P <0.05 and ^##^P <0.01 vs vehicle.
(TIF)
