# Supplementary material for: Effect of inhibition of CBP-coactivated β-catenin-mediated Wnt signalling in uremic rats with vascular calcifications
Source: PLoS One. 2018 Aug 3;13(8):e0201936. doi: 10.1371/journal.pone.0201936 (PMC6075782; doi:10.1371/journal.pone.0201936)
Supplement: S1 Table — In a previous study we performed RNA deep sequencing of aortae from Ctrl rats and uremic rats with vascular calcification [15]. The database was searched for genes related to Wnt-signaling; specifically, Wnt ligands, intracellular Wnt-signaling transducers, Wnt target genes and Wnt inhibitors were searched. Only genes with significant differences between uremic and Ctrl rats are presented. (PDF) [file pone.0201936.s001.pdf]

GENE	NAME	Ctrl (rpkm)	Uremic (rpkm)	Log2(Uremic/Ctrl)	P
**WNT LIGANDS**					
**Wnt16**	wingless-type MMTV integration site family, member 16	0.5	4.2	3.2	0.0048
**Wnt5b**	wingless-type MMTV integration site family, member 5B	7.1	2.1	-1.7	0.0017
**WNT SIGNALING**					
**Fzd2**	frizzled family receptor 2	35	23	-0.6	0,0064
**Fzd1**	frizzled family receptor 1	9.1	19	1.0	0,0007
**Fzd4**	frizzled family receptor 4	24	16	-0.5	0,02
**Dvl1**	dishevelled segment polarity protein 1	37	27	-0.5	0.04
**WNT TARGET GENES**					
**Jun**	jun proto-oncogene	216	66	-1.7	0,0007
**Ccnd1**	cyclin D1	21	86	2.1	0,0007
**Myc**	myc proto-oncogene	9.5	20	1.1	0.0007
**Snai1**	snail1	17	53	1.6	0.0007
**Fn1**	fibronectin 1	192	3778	4.3	0.0007
**Bmp4**	bone morphogenetic protein 4	40	80	1.0	0.0007
**Runx2**	runt-related trancription factor 2	1.3	6.0	2.2	0.0007
**Fst**	follistatin	3.8	13	1.8	0.0007
**Grem1**	gremlin 1	1.4	5.5	2.0	0.0007
**Grem2**	gremlin 2	12	2.8	-2.0	0.0007
**Postn**	periostin	87	810	3.2	0.0007
**WNT INHIBITORS**					
**Sost**	sclerostin	12	139	3.6	0.0007
**Sfrp4**	secreted frizzled-related protein 4	17	39	1.2	0.0007
**Frzb**	frizzled-related protein	806	442	-0.9	0.003
