# Supplementary material for: Effect of inhibition of CBP-coactivated β-catenin-mediated Wnt signalling in uremic rats with vascular calcifications
Source: PLoS One. 2018 Aug 3;13(8):e0201936. doi: 10.1371/journal.pone.0201936 (PMC6075782; doi:10.1371/journal.pone.0201936)
Supplement: S2 Table — Data is presented as mean ± SD and PTH as median and [range]. n = 6–9. Vehicle-treated Ctrl, CRF and CRF-D rats were compared by one-way ANOVA and Dunnets multiple comparison with *P<0.05, **P<0.001 and ***P<0.0001 vs Ctrl. ICG-001- and vehicle-administered groups were compared using unpaired two-tailed t-test with #P <0.05 vs vehicle. (PDF) [file pone.0201936.s002.pdf]

		Ctrl	CRF	CRF-D	CRF-D, 8w	ANOVA
**Bodyweight, g**	Vehicle	243±11	225±23	231±28	201±37**	P<0.05
	ICG	245±28	237±19	243±22		
**Urea, mmol/L**	Vehicle	4.9±1.2	11.4±1.6*	12.5±5.6**	13.3±8.7*	P<0.05
	ICG	5.7±0.9	10.1±2.3	9.3±1.0		
**Creatinine, µmol/L**	Vehicle	27±2	50±6**	53±12***	58±19***	P<0.0001
	ICG	27±3	56±10	54±10		
**Phosphate, mmol/L**	Vehicle	1.89±0.41	1.59±0.06	1.91±0.10	2.12±0.21	P<0.01
	ICG	1.70±0.35	2.20±0.44 [#]	1.97±0.18		
**Total Ca, mmol/L**	Vehicle	2.20±0.14	2.37±0.08	2.37±0.11	2.41±0.21*	P<0.05
	ICG	2.23±0.09	2.25±0.12	2.27±0.14		
**Ca^{2+}, mmol/L**	Vehicle	1.45±0.08	1.36±0.12	1.40±0.16	1.60±0.18	P<0.05
	ICG	1.44±0.13	1.37±0.07	1.36±0.12		
**PTH, pg/mL**	Vehicle	209 [105-299]	342 [296-670]*	474 [287-583]	17 [17-69]	P<0.001
	ICG	276 [77-386]	1227 [467-1836]	693 [247-1397]		
**Intact FGF23, pg/mL**	Vehicle	412±95	1371±284*	1994±491**	42028±37101***	P<0.001
	ICG	284±68[#]	1901±926	2414±1307		
