# Supplementary material for: Effect of inhibition of CBP-coactivated β-catenin-mediated Wnt signalling in uremic rats with vascular calcifications
Source: PLoS One. 2018 Aug 3;13(8):e0201936. doi: 10.1371/journal.pone.0201936 (PMC6075782; doi:10.1371/journal.pone.0201936)
Supplement: S3 Table — (PDF) [file pone.0201936.s003.pdf]

Gene symbol	Name		Sequence
**Actb**	Actin, beta	F<br>R	**5'**-GAACCCTAAGGCCAACCGTGAA-**3'**<br>**5'**-GCGCGTAACCCTCATAGATG-**3'**
**Ccnd1**	CyclinD1	F<br>R	**5'**-CCTCTCCTGCTACCGCACAA-**3'**<br>**5'**-CGCAGGCTTGACTCCAGAAG-**3'**
**Ctnnb1**	Catenin, beta	F<br>R	**5'**-TCCAGGAATGAAGGCGTGGCAACA-**3'**<br>**5'**-CAGTCCGAGATCAGCAGTCTCA-**3'**
**Fn1**	Fibronectin 1	F<br>R	**5'**-GGGCGAAGGCAATGGGCGTATCA-**3'**<br>**5'**-CTGGGGGTGGGTCTGGGGTTGGTA-**3'**
**Hprt**	Hypoxantine phosphoribosyltransferase 1	F<br>R	**5'**-CCCAGCGTCGTGATTAGTGA-**3'**<br>**5'**-CCAAATCTTCAGCATAATGATTAGGTAT-**3'**
**Inhba**	Inhibin beta A subunit	F<br>R	**5'**-AGTGGGGGAAAACGGGTATGTGGA-**3'**<br>**5'**-CCTGTTGGCCTTGGGGACTTTCA-**3'**
**Jun**	Jun	F<br>R	**5'**-GGGGGAGCACTGCGGTCTGGAG-**3'**<br>**5'**-GAGGCGTTGAGGGCATCGTCGTAG-**3'**
**Postn**	Periostin	F<br>R	**5'**-GAACCCGGAGTCACCAACATC-**3'**<br>**5'**-TGCTGCCACGAACGAACTTA-**3'**
**Rplp0**	Ribosomal protein lateral stalk subunit P0 (Arbp)	F<br>R	**5'**-AAAGGGTCCTGGCTTTGTCT-**3'**<br>**5'**-GCAAATGCAGATGGATCG-**3'**
**Rpl13a**	Ribosomal protein L13A	F<br>R	**5'**-CCCTCCACCCTATGACAAGA-**3'**<br>**5'**-CCTTTTCCTTCCGTTTCTCC-**3'**
**Runx2**	Runt-related transcription factor 2	F<br>R	**5'**-TCCATTCCACCACGCCGCTGTCTT-**3'**<br>**5'**-GCCTGGGAACTGCCTGGGGTCTGA-**3'**
**Snai1**	Snail1	F<br>R	**5'**-ATATTGTAATAAGGAGTACCTCA-**3'**<br>**5'**-AGGCCTGACACTGGTATCTCTTC-**3'**
**Snai2**	Snail2	F<br>R	**5'**-ACGCCTCCAAGAAGCCCAACTAC-**3'**<br>**5'**-TTACACGCCCCAAAGATGAGGA-**3'**
**Sost**	Sclerostin	F<br>R	**5'**-GCCTCCTCAGGAACTAGAGAAC-**3'**<br>**5'**-TACTCGGACACGTCTTTGGTG-**3'**
**Tgfb1**	Transforming growth factor, beta 1	F<br>R	**5'**-TCCACGTGGAAATCAATGGGATCA-**3'**<br>**5'**-CAGTTCTTCTCTGTGGAGCTGAA-**3'**
**Tnfsf11**	Rankl	F<br>R	**5'**-CGAGCGCAGATGGATCCTAACAGA-**3'**<br>**5'**-TCCCTTTGCACGGCCCCTTGAA-**3'**
**Vim**	Vimentin	F<br>R	**5'**-CGTGATGTCCGCCAGCAGTAT-**3'**<br>**5'**-TGGCGCAGGGCATCGTTGTT-**3'**
