# Supplementary figures and images for: Effect of inhibition of CBP-coactivated β-catenin-mediated Wnt signalling in uremic rats with vascular calcifications
Source: PLoS One. 2018 Aug 3;13(8):e0201936. doi: 10.1371/journal.pone.0201936 (PMC6075782; doi:10.1371/journal.pone.0201936)

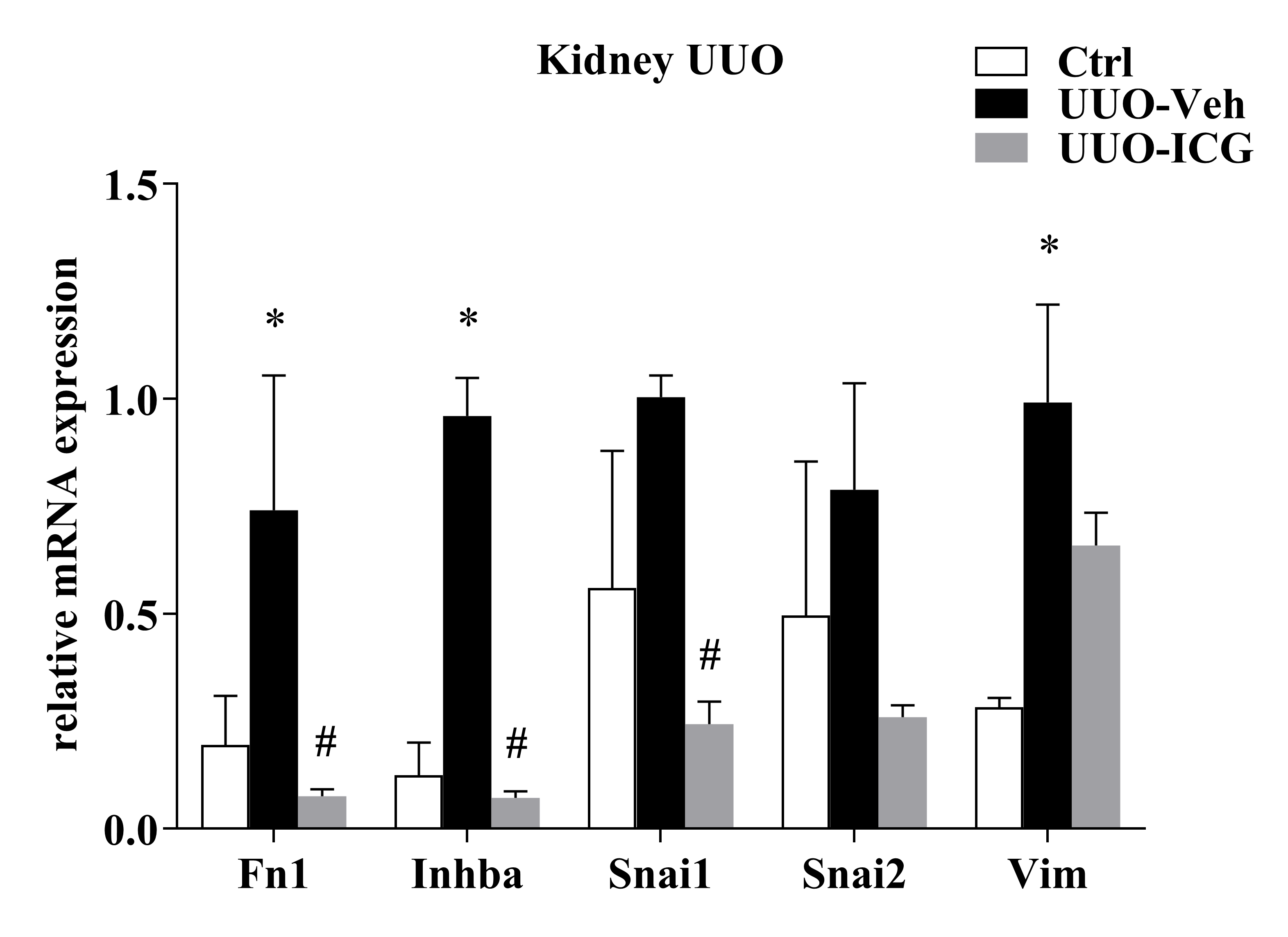

Supplement: S1 Fig — ICG-001 was administered at the time of obstruction and subsequently daily at a dose of 5mg/kg. Rats were sacrificed after three days of UUO. Kidney gene expression was examined in the obstructed kidney from ICG-001 (UUO-ICG) and vehicle treated rats (UUO-Veh) as well as in the normal kidney from untouched control rats (Ctrl). UUO resulted in an induction in kidney expression of profibrotic genes, and ICG-001 administration ameliorated this response. Data is presented as mean ± SD. Data is presented as mean ± SD. n = 6–9. *P<0.05 vs Ctrl by unpaired two-tailed t-test, #P <0.05 vs vehicle by unpaired two-tailed t-test. (TIF) [file pone.0201936.s004.tif]

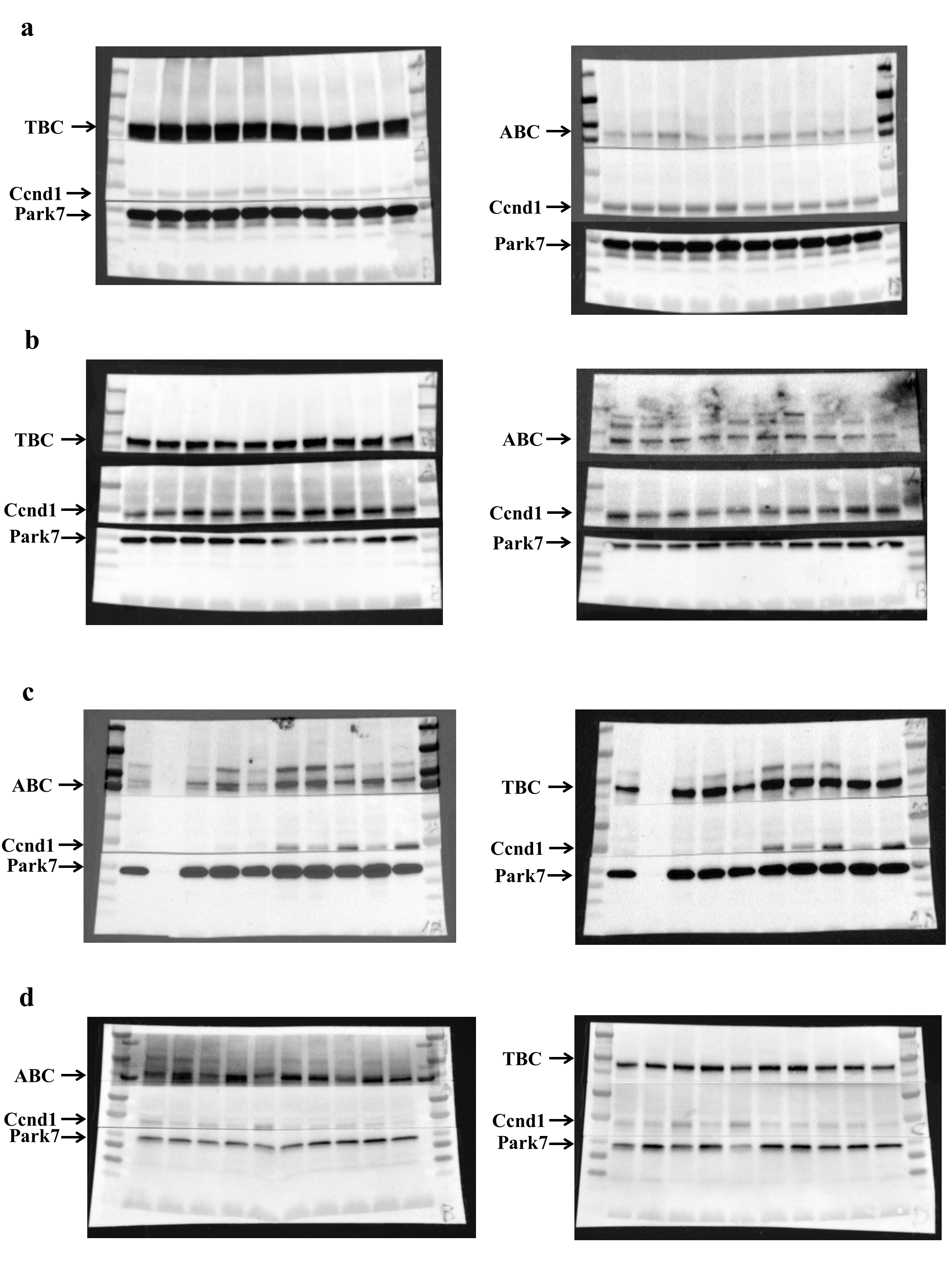

Supplement: S2 Fig — (a) WB from Fig 3B, (b) WB from Fig 3D, (c) WB from Fig 4E, (d) WB from Fig 4F. (TIF) [file pone.0201936.s005.tif]

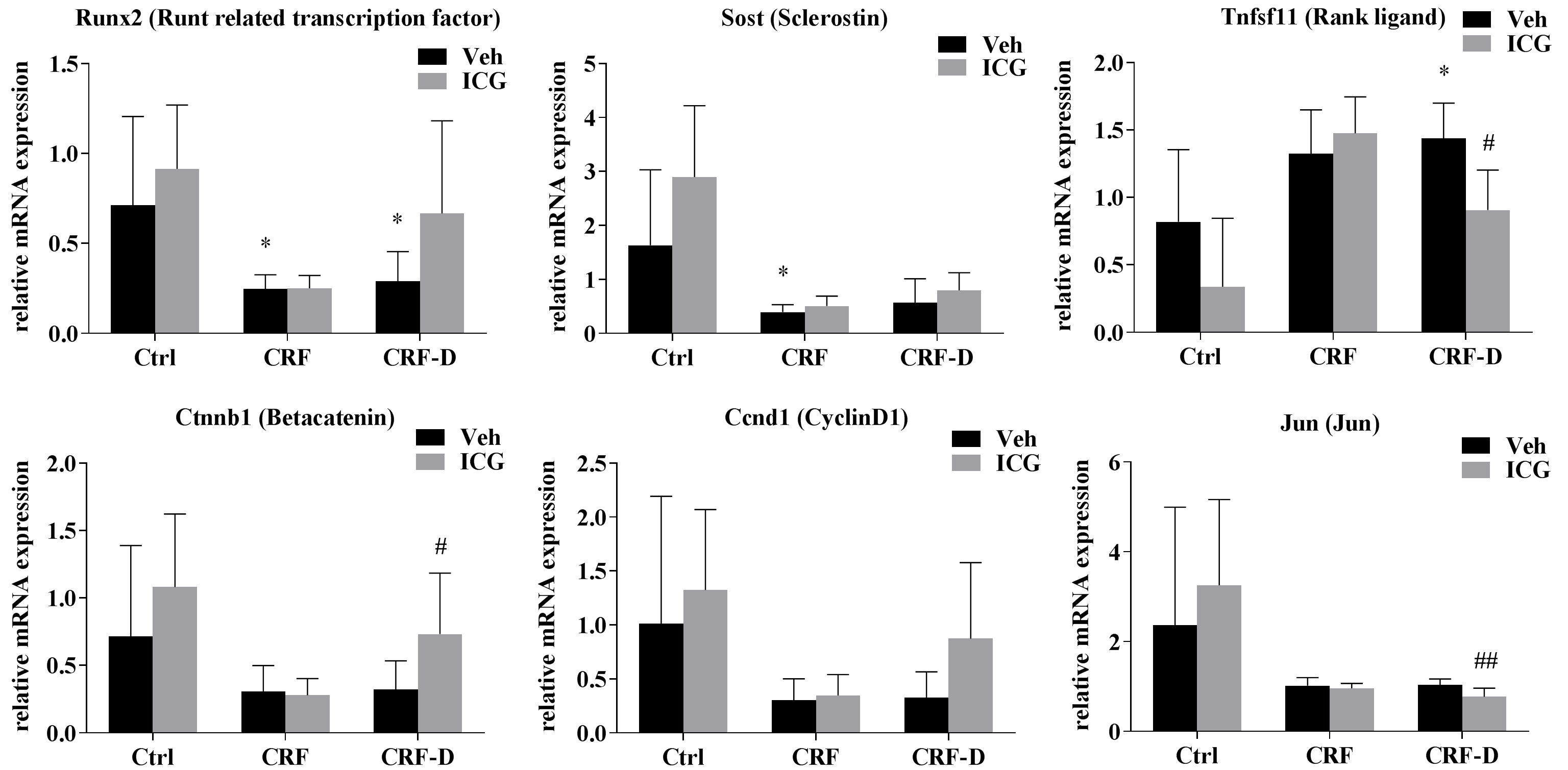

Supplement: S3 Fig — Gene expression was examined in cortical femoral bone tissue by qPCR. Gene expression of the early osteoblast marker Runx2 and the mature osteocyte marker Sost was decreased in CRF and CRF-D rats compared to Ctrl, whereas the osteoclast differentiation marker Rankl was increased in CRF-D rats compared to Ctrl. ICG-001 treatment resulted in a decrease in the expression of Rankl in CRF-D rats, and surprisingly an increase in Ctnnb1 and a decrease in Jun was seen in ICG-001 treated CRF-D rats compared to vehicle-treated CRF-D rats. Data is presented as mean ± SD. n = 6–9. Vehicle-treated Ctrl, CRF and CRF-D rats were compared by one-way ANOVA and Dunnets multiple comparison with *P<0.05 and **P<0.001 vs Ctrl. ICG-001- and vehicle-treated groups were compared using unpaired two-tailed t-test with #P <0.05 and ##P <0.01 vs vehicle. (TIF) [file pone.0201936.s006.tif]
